# A Low-Cost Rectal Foreign Body Removal Simulator

**DOI:** 10.5070/M5.56950

**Published:** 2026-10-31

**Authors:** Joshua Ginsburg, Vivek Medepalli, Monique Graf, Jason Fuller

**Affiliations:** * UT Southwestern, Department of Emergency Medicine, Dallas, TX; ^ Baylor Scott & White All Saints Medical Center, Department of Emergency Medicine, Dallas, TX; † Baylor Scott & White All Saints Medical Center-Fort Worth, Department of Emergency Medicine, Fort Worth, TX; ** Spartanburg Regional Health Systems, Department of Emergency Medicine, Spartanburg, SC

## Abstract

**Audience:**

This rectal foreign body removal model is designed to instruct emergency medicine residents. However, the innovation is appropriate for medical students, advanced practice providers (APPs), attending physicians, and clinicians in other medical specialties.

**Introduction:**

Rectal foreign bodies are relatively uncommon emergency department presentations but are associated with significant morbidity and mortality.^1^ These foreign bodies require prompt recognition and skilled management, which can be challenging due to stigma, patient discomfort, and procedural complexity. Learners often have limited opportunities for hands-on experience with rectal foreign body removal, and to our knowledge, no established simulation tool currently exists for training in this procedure. This task trainer was designed as an easily assembled, cost-effective simulation tool to support procedural practice and promote safe, effective performance when applied in the emergency department setting.

**Educational Objectives:**

This task trainer is intended to facilitate hands-on training in rectal foreign body removal. By the end of this training session, learners should be able to: 1) describe the indications and contraindications for rectal foreign body removal in the emergency department, and 2) demonstrate the ability to remove rectal foreign bodies of varying sizes, shapes, and depths of insertion using multiple techniques.

**Educational Methods:**

This innovation consists of a task trainer for the simulated removal of rectal foreign bodies. The tool was constructed using readily available, inexpensive materials to accommodate the removal of foreign bodies of varying sizes and depths, allowing for customizable scenarios of variable difficulty. The session began with a brief didactic overview covering anatomy, tools, procedural steps, and complications of rectal foreign body removal, followed by supervised hands-on practice using the model. Faculty provided individualized feedback to help learners refine their techniques.

**Research Methods:**

A pre- and post-activity survey was administered to participants to assess comfort with rectal foreign body removal and gather feedback on the educational session and task trainer. The pre-activity survey asked learners to report the number of rectal foreign body removals they had performed and to rate their comfort with the procedure on a 7-point Likert scale (1 = totally comfortable, 7 = totally uncomfortable). The post-activity survey included the same comfort scale to allow for comparison, along with additional items evaluating the model and session. Learners rated the model’s realism (1 = extremely realistic, 5 = not at all realistic), the degree to which the session increased their confidence performing the procedure (1 = extremely, 5 = not at all), and the overall usefulness of the model for teaching rectal foreign body removal (1 = extremely useful, 5 = not useful at all). This evaluation approach addressed the reaction and learning levels of the Kirkpatrick model.

**Results:**

Thirty-nine learners completed the pre-activity survey and 37 completed the post-activity survey. Most learners (79%) had never previously performed a rectal foreign body removal. Following the session, learners reported significant improvements in comfort with the procedure. The task trainer received favorable ratings for realism, educational usefulness, and its ability to build confidence in performing the procedure on real patients.

**Discussion:**

This educational session was both effective and well-received. Learners began with limited experience performing rectal foreign body removal, a procedure that may be infrequently encountered but carries significant risk. Providing a structured opportunity for supervised practice in a low-risk environment likely contributed to the observed improvements in reported comfort and confidence.

Beyond numerical gains, this session highlighted an important educational gap: most learners have limited exposure to this procedure that they are likely to encounter in their careers, underscoring the need for accessible methods of training in rare procedures. The favorable feedback regarding realism, confidence, and usefulness suggests that even a simple model can provide meaningful educational value.

The main takeaway is that this rectal foreign body removal task trainer offers a practical, reproducible approach to improving learner preparedness in managing a sensitive and technically challenging procedure in the emergency department setting.

**Topics:**

Rectal foreign bodies, medical simulation, hands-on training, emergency medicine education, rare procedures.

## USER GUIDE

**Table:** 

List of Resources:	
Abstract	1
User Guide	3
Appendix A: Facilitator’s Guide	7

**Learner Audience:**

This rectal foreign body removal model is designed to instruct emergency medicine residents. However, the innovation is appropriate for medical students, advanced practice providers (APPs), attending physicians, and clinicians in other medical specialties.

**Time Required for Implementation:**

Each model takes less than 5 minutes to make. If the disposable sock is damaged during the procedure, it can be replaced and retaped in about 1 minute. Learners should spend between 5 and 15 minutes using this task trainer to allow time to practice multiple approaches on foreign bodies of various sizes, shapes, and depths of insertion.

**Recommended Number of Learners per Instructor:**

We recommend a ratio of 3–5 learners per instructor.

**Topics:**

Rectal foreign bodies, medical simulation, hands-on training, emergency medicine education, rare procedures.

**Objectives:**

By the end of this session, learners will be able to:

1. Describe the indications and contraindications for rectal foreign body removal in the emergency department.
2. Demonstrate the ability to remove rectal foreign bodies of varying sizes, shapes, and depths of insertion using multiple techniques.

### Linked objectives, methods and results

The objectives of this session were addressed through an intentionally structured combination of instruction, hands-on practice, and debriefing. The initial faculty-led discussion reviewed indications, contraindications, and procedural considerations, ensuring learners began with a shared foundational knowledge before practice.

The hands-on portion using the task trainer allowed learners to apply concepts immediately, practicing foreign body removal techniques with real-time faculty feedback. This approach supported skill development with individualized guidance in a low-risk environment.

Finally, a debrief was conducted to reinforce key learning points and clarify procedural steps. This sequence of instruction, practice, and debriefing was selected because it efficiently combines knowledge acquisition and skill application within a single, brief educational session.

### Recommended pre-reading for instructor

- Rectal foreign bodies: Not always a simple ED diagnosis. emDocs. https://www.emdocs.net/rectal-foreign-bodies-not-always-a-simple-ed-diagnosis.
- Nesemann S, Hubbard KA, Siddiqui MI, Fernandez WG. Rectal foreign body removal in the emergency department: A case report. *Clin Pract Cases Emerg Med*. 2020;4(3):450–453. doi:10.5811/cpcem.2020.7.47237
- Moderated by Madbak F, Valdez C. Traumacast Episode 95: Anorectal emergencies. Eastern Association for the Surgery of Trauma (EAST). https://www.east.org/education-resources/traumacasts/detail/144/anorectal-emergencies-

### Learner responsible content (LRC)

- We did not require residents to do any pre-reading. However, any or all of the instructor pre-reading above would be appropriate learner responsible content.

### Implementation Methods

The session should begin with a faculty-led briefing covering indications and contraindications for rectal foreign body removal in the emergency department. Special emphasis should be placed on identifying objects that pose a high risk of perforation or require removal under general anesthesia in the operating room. Faculty will then review the necessary materials for local anesthesia, proper patient positioning, and appropriate removal techniques.

Learners will individually practice removing foreign bodies of varying sizes, shapes, and depths of insertion using the task trainer, rotating every 5–15 minutes. Potential foreign bodies for practice include cucumbers, bananas, lipstick tubes, lightbulbs, and golf balls. Faculty will provide real-time guidance and feedback throughout the activity, followed by a brief debriefing session summarizing key procedural indications, contraindications, technical steps, and learner reflections on the session.

### List of items required to replicate this innovation

1. Shipping boxes, approximately 10” x 7” x 5” ($14.99 for a pack of 6 at https://www.amazon.com/dp/B0CTMPRSZW?ref=ppx_pop_mob_ap_share&th=1)
2. Box cutter ($7.99 for a pack of 2 at https://www.amazon.com/dp/B08NX1XYBN?ref=cm_sw_r_cso_em_apin_dp_P6G1GPRNFCCKZ0ZFSJEC&ref_=cm_sw_r_cso_em_apin_dp_P6G1GPRNFCCKZ0ZFSJEC&social_share=cm_sw_r_cso_em_apin_dp_P6G1GPRNFCCKZ0ZFSJEC&titleSource=avft-a&previewDoh=1&previewDohDeal=1&th=1)
3. Foam tape, 4” width or larger ($13.48 for 5.5 yards at https://www.amazon.com/dp/B00ZVVF29C?ref=ppx_pop_mob_ap_share)
4. Disposable try on socks ($13.95 for a pack of 144 socks at https://www.amazon.com/dp/B07Y3V45CW?ref=ppx_pop_mob_ap_share)
5. Foreign bodies of various sizes and shapes, such as cucumbers, bananas, lipstick tubes, lightbulbs, and golf balls, depending on what is freely available
6. Tools to aid in visualization and removal of the foreign body, including an anoscope, pediatric vaginal speculum, ringed forceps, and Foley catheters (all available in most emergency departments)

### Approximate cost of items to create this innovation

The cost of all items used to create this task trainer was $50.41. The approximate cost per model, including 1 shipping box, 1 box cutter, 1 disposable sock, and 1 foot of foam tape, was $7.41.

### Detailed methods to construct this innovation

1. Using a box cutter, cut a 3-inch diameter hole in the front of a shipping box, keeping the top of the box untaped to allow for visualization from above.
  **Figure:**
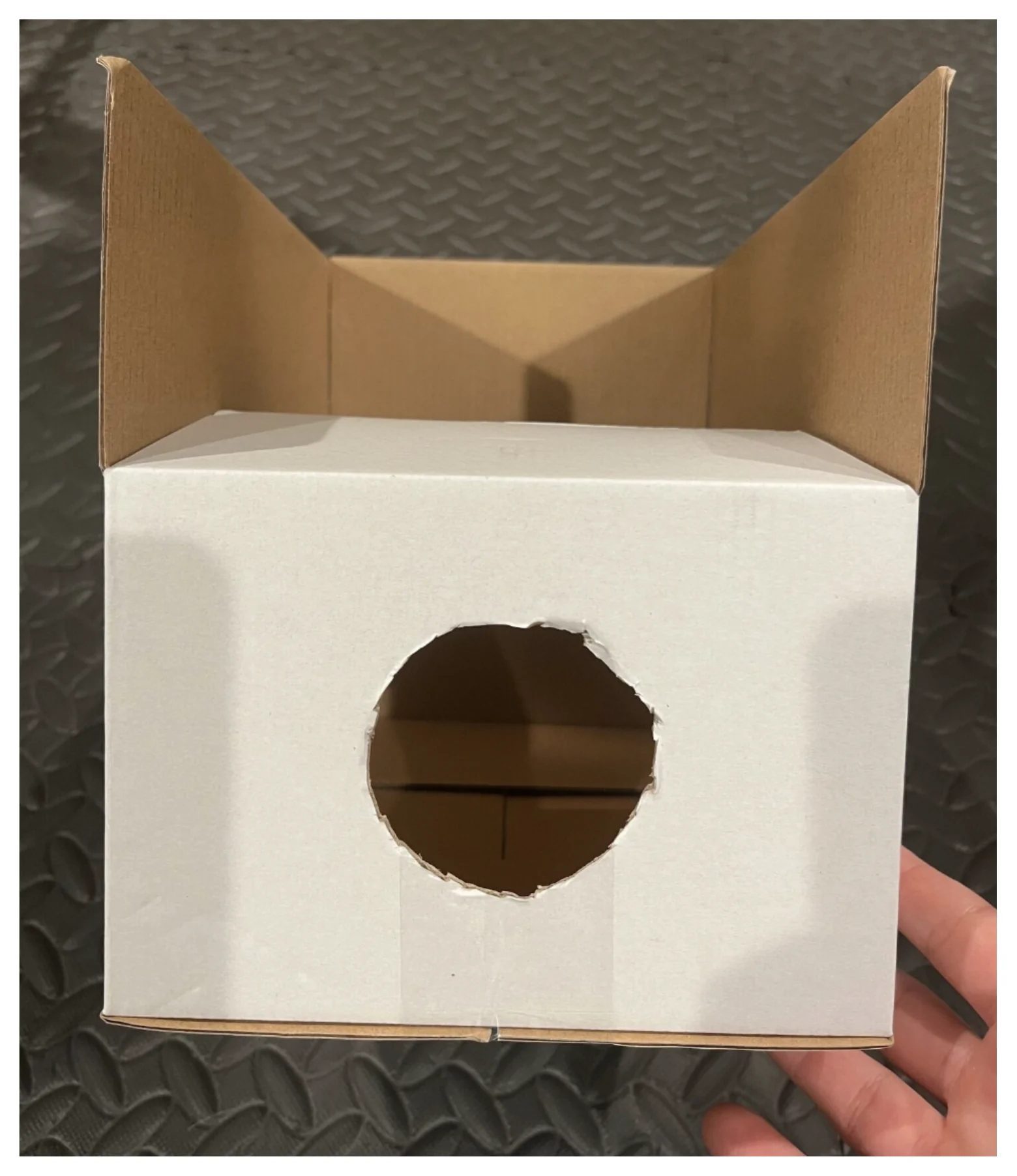
Box With Hole: Author’s own image.
2. Insert a disposable sock through the hole, stretching the open end of the sock around the hole while the closed end hangs in the interior of the box.
  **Figure:**
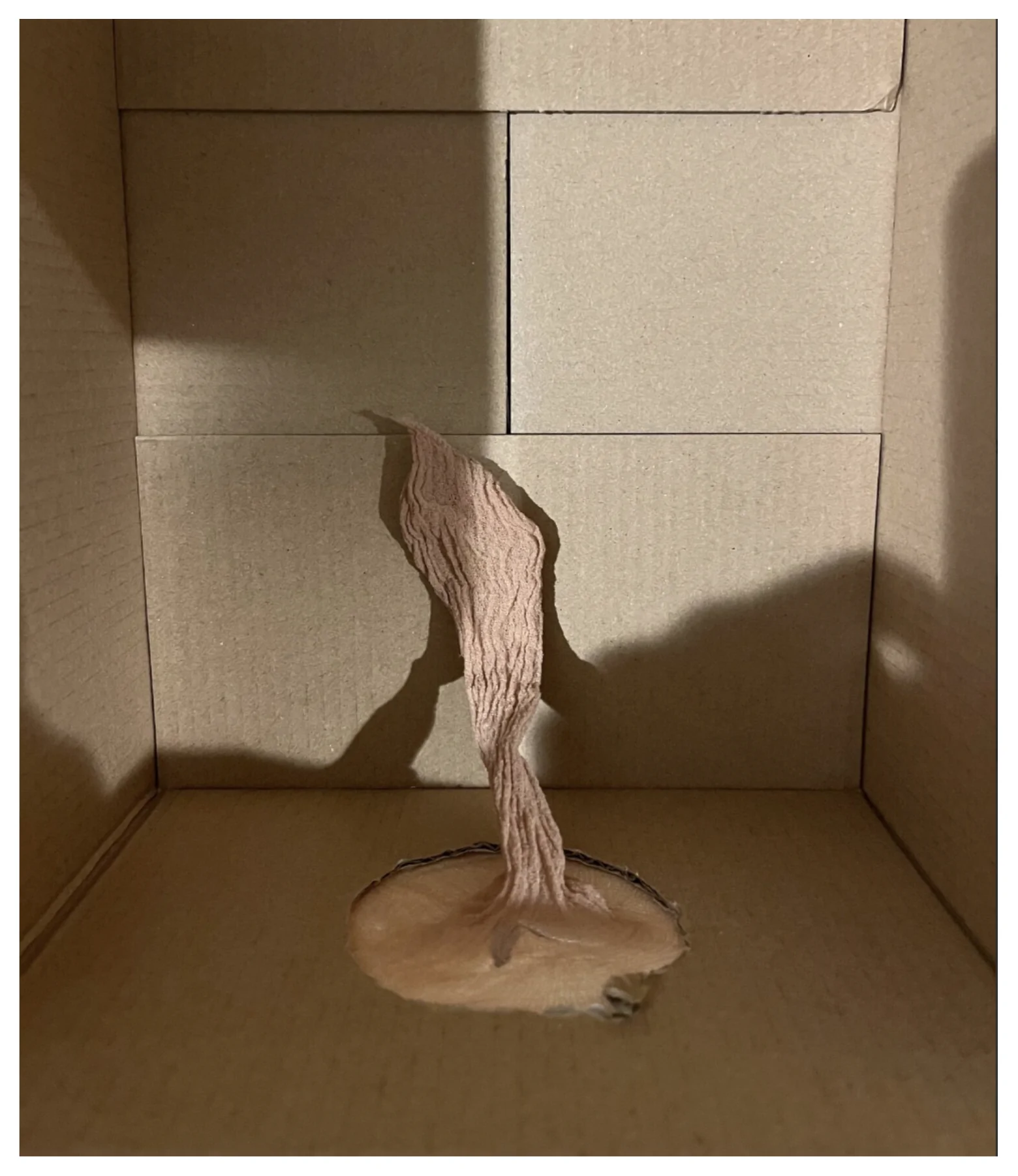
Sock Inserted: Author’s own image.
3. Using foam tape, tape the open end of the sock in place around the hole. This step may require one person to hold the sock in place while another applies the tape.
  **Figure:**
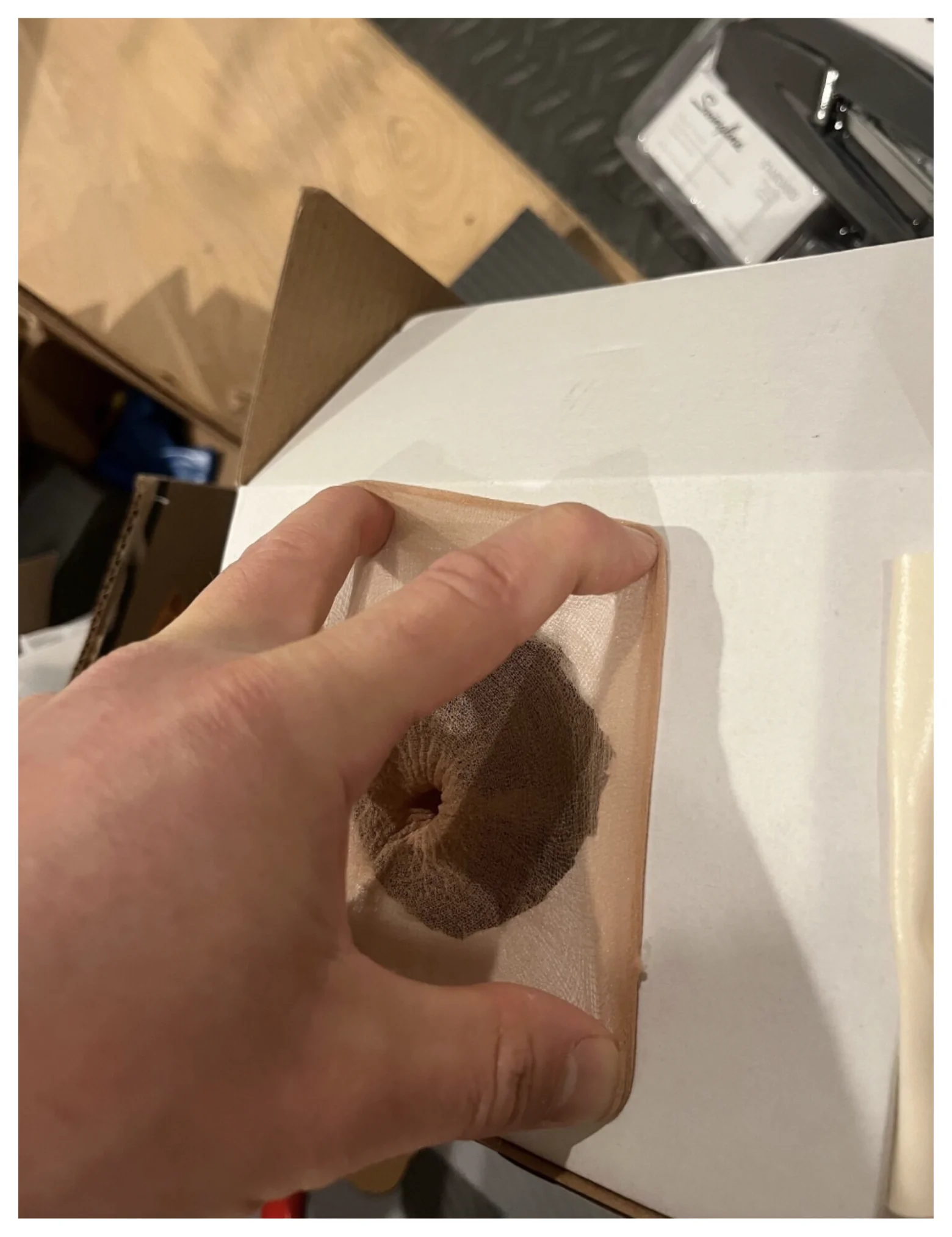
Sock Taped: Author’s own image.
4. Using a box cutter, make a cruciate incision in the shape of a plus sign in the foam tape, taking care not to cut the sock.
  **Figure:**
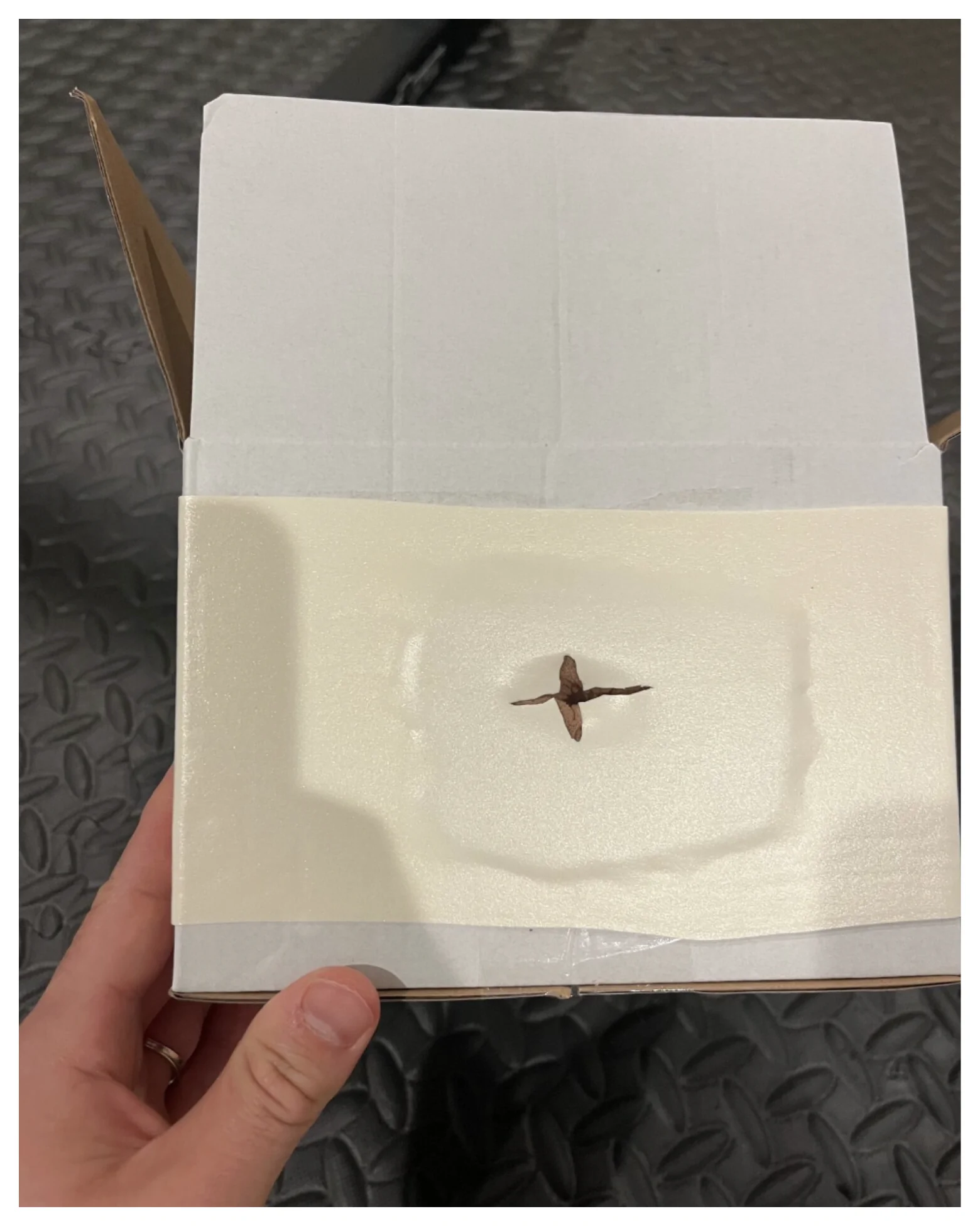
Tape Placed and Cut: Author’s own image.
5. Gently insert foreign bodies through the cruciate incision into the sock to the desired depth. The instructor may have to hold tension on the sock as shown.
  **Figure:**
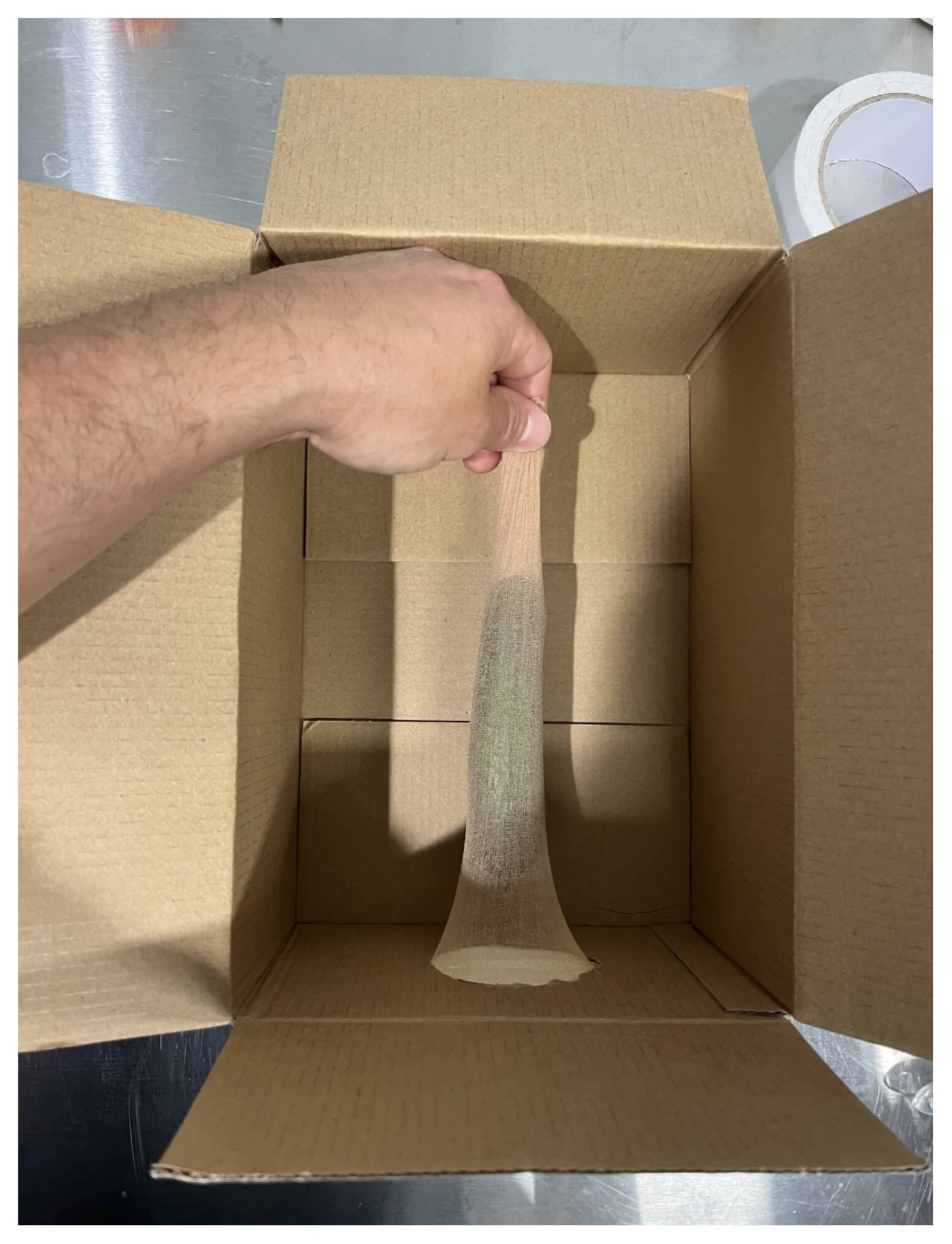
Foreign Body Inserted: Author’s own image.

### Results and tips for successful implementation

We tested this on learners during our Advanced Resuscitation Day, a yearly conference day during which residents move in small groups between stations to practice rare and high-acuity procedures. However, this easily assembled, portable task trainer could be used for training in multiple educational environments, including during a lecture on the topic or as just-in-time training prior to performing the procedure.

We administered a pre- and post-activity survey via REDCap to participants to assess comfort with rectal foreign body removal and gather feedback on the educational session and task trainer. A total of 39 learners completed the pre-activity survey, and 37 completed the post-activity survey. Prior to the session, 79% of learners had never removed a rectal foreign body, while 21% had removed one. Before the hands-on activity, 57% of learners reported being uncomfortable with rectal foreign body removal (3% totally uncomfortable, 23% very uncomfortable, 31% somewhat uncomfortable); 23% were somewhat comfortable, and none were very or totally comfortable.

Following the session, 84% of learners reported being comfortable with the procedure (49% somewhat comfortable, 30% very comfortable, 5% totally comfortable), and none reported being uncomfortable.

Regarding the model itself, 11% of learners rated it as extremely realistic, 5% very realistic, 62% moderately realistic, and 22% slightly realistic; none rated it as not realistic. Learners found the model useful for teaching rectal foreign body removal, with 16% rating it extremely useful, 57% very useful, 16% moderately useful, and 11% slightly useful; none rated it as not useful.

When asked how much practicing with the model increased their confidence in performing the procedure on a real patient, 5% responded extremely, 60% considerably, 24% moderately, and 11% slightly; none selected not at all.

We did not make any modifications to the task trainer or the educational methods, since survey results and informal feedback were positive. However, future iterations might improve realism through modifications such as using a more anatomically contoured outer structure, additions such as pressure bags or blood pressure cuffs to increase pressure on the simulated rectal vault or adding a tissue-like liner inside the sock to more closely approximate mucosal texture.

### Associated content

- Appendix A: Facilitator’s Guide

## INNOVATION MATERIALS

### Appendix A Facilitator’s Guide

The following guide was discussed with the learners prior to practice using the task trainer.

#### Initial Considerations^2^

- Good history and physical
  ○ Consider what material you’re working with or how large the object might be
    ▪ Ensure the object is not sharp before performing a digital rectal exam
  ○ Examine the abdomen for signs of peritonitis
- Imaging
  ○ Abdominal X-ray, Computed tomography of the abdomen and pelvis (CT A/P)
  ○ Evaluate for location of object as well as for perforation
    ▪ If the object is high-lying, manual extraction is likely futile and patient will probably need the operating room (OR) to have anus dilated under anesthesia
- Consultation
  ○ Consider surgical consultation in the event of unsuccessful removal attempts, if the object has the potential to injure the anal sphincter, in the case of sharp objects, of if there is high risk of ischemia/perforation with removal
  ○ Consider GI consultations if sigmoidoscopy may be indicated after removal, such as in the case of prolonged retention or if an object with sharp corners is removed

#### Materials Required^2^–^4^

- Lubricant
- Ring Forceps
- Light Source
- +/− Anoscope
- +/− Pediatric Vaginal Speculum
- Perianal Nerve Block (Ring Block)
  ○ Lidocaine
  ○ 23–25 gauge (G) Needle
  ○ 10 cubic centimeter (cc) Syringe

#### Procedural Steps^2^–^4^

1. Place the patient into dorsal lithotomy position for optimal effect. You can attempt digital rectal examination with lubrication or visualization with anoscope to attempt to deliver object; however, there is a risk of pushing object further up.
2. Perform a perianal nerve block by injecting lidocaine in a ring fashion around the anus to assist in relaxing the sphincter. Additionally, anxiolysis with benzodiazepines, analgesia with opioids, and/or procedural sedation should be considered.
3. Place large amount of surgical lubricant at the anal introitus to facilitate passage of object once grasped
4. Insert ring forceps and slowly expand to dilate anus and grasp object. Once grasped, retract towards the introitus with mild-moderate pressure in a steady, constant fashion. Addition of suprapubic pressure and patient Valsalva may help facilitate removal of the object.
  ○ Note: Large bulbar objects can create a vacuum effect on the anal mucosa. In this case, make use of a Foley Catheter!
    - Insert Foley to slide around object and inflate balloon proximal to it. Can use multiple catheters on all sides of object.
    - With balloon inflated, retract foley catheter(s) towards the introitus to deliver the object.
5. Consider complications, including laceration, hematoma, bowel perforation, and damage to the anal sphincter.
